# Nanoparticles to Target and Treat Macrophages: The Ockham’s Concept?

**DOI:** 10.3390/pharmaceutics13091340

**Published:** 2021-08-26

**Authors:** Mireia Medrano-Bosch, Alazne Moreno-Lanceta, Pedro Melgar-Lesmes

**Affiliations:** 1 Department of Biomedicine, School of Medicine, University of Barcelona, Casanova 143, 08036 Barcelona, Spain; mireiamb_7@hotmail.com (M.M.-B.); amorenol@clinic.cat (A.M.-L.); 2 Biochemistry and Molecular Genetics Service, Hospital Clínic Universitari, IDIBAPS, CIBERehd, 08036 Barcelona, Spain; 3 Institute for Medical Engineering and Science, Massachusetts Institute of Technology, Cambridge, MA 02139, USA

**Keywords:** macrophages, nanoparticles, inflammation, cancer, regeneration

## Abstract

Nanoparticles are nanomaterials with three external nanoscale dimensions and an average size ranging from 1 to 1000 nm. Nanoparticles have gained notoriety in technological advances due to their tunable physical, chemical, and biological characteristics. However, the administration of functionalized nanoparticles to living beings is still challenging due to the rapid detection and blood and tissue clearance by the mononuclear phagocytic system. The major exponent of this system is the macrophage. Regardless the nanomaterial composition, macrophages can detect and incorporate foreign bodies by phagocytosis. Therefore, the simplest explanation is that any injected nanoparticle will be probably taken up by macrophages. This explains, in part, the natural accumulation of most nanoparticles in the spleen, lymph nodes, and liver (the main organs of the mononuclear phagocytic system). For this reason, recent investigations are devoted to design nanoparticles for specific macrophage targeting in diseased tissues. The aim of this review is to describe current strategies for the design of nanoparticles to target macrophages and to modulate their immunological function involved in different diseases with special emphasis on chronic inflammation, tissue regeneration, and cancer.

## 1. Introduction

**Figure 1:**
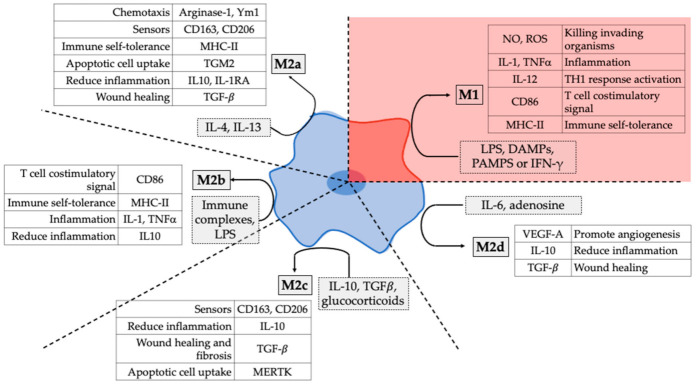
Overview of macrophage phenotypes. The figure shows the phenotypical division of macrophages in M1 and M2 phenotype and the subcategories of M2 macrophages (M2a, M2b, M2c, M2c). It also shows the markers expressed by the different macrophage phenotypes upon activation by different stimuli and their functions. CD: Cluster of differentiation; LPS: lipopolysaccharide; MHC-II: Major histocompatibility complex—II; TGM2: Transglutaminase 2 (TGM2); IL: Interleukin; IL-1RA: interleukin 1 receptor antagonist; TGF-β: tumor growth factor beta; TNFα: tumor necrosis factor alpha; ECM: extracellular matrix; MERTK: MER Proto-Oncogene Tyrosine Kinase; NOS: Nitrogen oxide species; ROS: Reactive oxygen species; DAMP: damage-associated molecular patterns; PAMP: pathogen molecular patterns; IFN-γ: Interferon gamma; VEGF-A: Vascular endothelial growth factor.

Macrophages are plastic cells from the innate immune system that play different roles in the development, homeostasis, tissue repair, and immune response [1]. They are found in all tissues where they display heterogeneous phenotypes depending on the tissue-specific function and the relationship with other parenchymal and non-parenchymal cells. In physiological conditions, they are responsible for key homeostatic processes such as the clearance of senescent cells and toxic materials, and the regulation of tissue metabolism [2]. Macrophages are the guardians in virtually all tissues of the body. Indeed, they are considered a hallmark of the response to foreign bodies. Their phagocytic capacity and the expression of a myriad of receptors on their surface allows a rapid response to changes in the local microenvironment [3]. They initiate an inflammatory response in response to tissue damage or invading organisms via stimulation and activation of lymphocytes and other immune cells to contain tissue injury or to disturb and destroy the pathogen [3]. Macrophages are traditionally divided into pro-inflammatory (M1) and anti-inflammatory or pro-regenerative (M2) phenotypes [4]. The local tissue microenvironment determines the macrophage polarization phenotype, M1-like or M2-like, in such a way that populations of both subsets can be found simultaneously coexisting in the same tissue. Macrophages are classically activated into the pro-inflammatory M1 phenotype in response to inflammatory stimuli such as lipopolysaccharides (LPS) or interferon-γ (IFN-γ). On the other hand, interleukin-4 (IL-4) and IL-13 alternatively activate the macrophage polarization to an anti-inflammatory M2 phenotype [3]. Polarized macrophages can be also reprogrammed by the combination of different agents promoting a phenotype reversion [5]. The different macrophage phenotypes play different roles in immune regulation, inflammation, tissue remodeling, proliferation, and metabolism, and their balance is critical to prevent disease and maintain immune system homeostasis [5]. Although macrophages are traditionally divided into M1 and M2 phenotypes, this division is way more complex. Some studies report that M2 macrophages can be further sub-categorized into distinct phenotypes: M2a, M2b, M2c, and M2d, which have a different transcriptional profile and different specific functions [6,7,8] (Figure 1).

**Figure 2:**
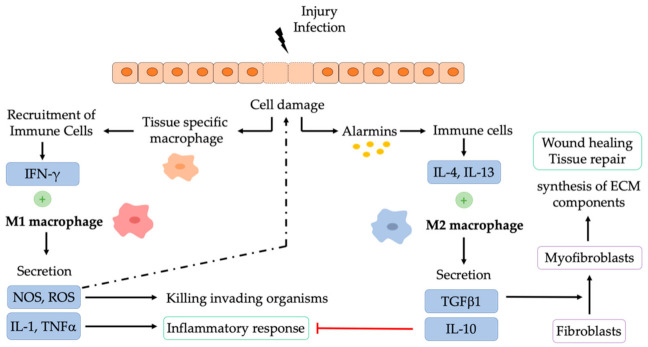
Macrophage activation by injury or infection. Cell damage triggers the activation of the immunological response, which is characterized by M1 macrophage accumulation. M1 macrophages trigger pro-inflammatory signals that are balanced by M2 macrophages (those that mediate anti-inflammatory responses and wound healing). IL: Interleukin; TGF-β: tumor growth factor beta; IFN-γ: interferon gamma; TNFα: tumor necrosis factor alpha; NO: Nitrogen oxide; ROS: Reactive oxygen species.

Damaged cells release specific molecules known as damage-associated molecular patterns (DAMPs) that activate the immune system in an analogous manner to pathogen-associated molecular patterns (PAMPs), small molecular motifs released from bacteria or viruses [9]. These endogenous molecules (calcium-binding proteins, structural and extracellular matrix proteins) display physiological functions in cells, but are recognized as danger signals when released into the extracellular space leading to downstream inflammation. Therefore, tissue-specific macrophage subpopulations detect signals that are not found in healthy tissues following infection or injury and recruit monocytes that differentiate into macrophages [3]. DAMPs, PAMPS, or IFN-γ secreted by lymphocytes induce the pro-inflammatory M1 macrophage phenotype [5]. M1 macrophages secrete a variety of pro-inflammatory mediators such as IL-1 and tumour necrosis factor (TNFα) that stimulate inflammation, and IL-12, which activates T helpers 1 (TH1), initiating the adaptive immune response [3]. M1 macrophages also secrete reactive oxygen species (ROS) and nitrogen species that contribute to the elimination of invading organisms (Figure 2). During this process, they also trigger substantial collateral tissue damage to the host. To prevent further tissue damage due to the inflammatory macrophage response, macrophages undergo apoptosis or polarization to an anti-inflammatory and pro-regenerative phenotype that dampens the pro-inflammatory response and facilitates wound healing [1]. Damaged epithelial cells release alarmins, which induce IL-4 and IL-13 secretion by other immune cells. IL-4 and IL-13 alternatively activate the macrophage polarization to an anti-inflammatory M2 phenotype [3]. M2 macrophages secrete anti-inflammatory cytokines such as IL-4, IL-13, or IL-10 to dampen the proinflammatory response [3] and specific and numerous growth factors such as transforming growth factor (TGFβ1) and vascular endothelial growth factors (VEGFs) to promote cell proliferation and angiogenesis [10]. TGFβ1 induces fibroblast differentiation to myofibroblasts to facilitate wound contraction and closure, as well as the synthesis of extracellular matrix (ECM) components (Figure 2) [11]. TGFβ1 also enhances the expression of tissue inhibitors of metalloproteinases (TIMP) to prevent ECM degradation. M2 macrophages can also regulate the proliferation and expansion of neighboring parenchymal and stromal cells and the activation of stem cells and local progenitor cell populations that participate in repair, thus activating wound healing and tissue growth to replace damaged areas [10].

**Figure 3:**
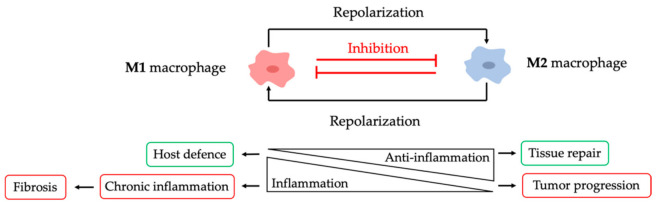
Macrophage roles on host defense. Macrophages are commonly divided into two phenotypes: M1 and M2 macrophages. These phenotypes are reversible, and macrophages can be re-programmed depending on the local stimuli. The inflammatory and anti-inflammatory responses carried out by macrophages need to be well balanced to prevent disease. Uncontrolled M1 macrophage activation can lead to chronic inflammation and un-controlled M2 macrophage activation can lead to tumour progression.

The inflammatory and anti-inflammatory responses orchestrated by macrophages need to be accurately regulated to prevent disease. Cytokines are the signals that mediate the coordination between immune cells to harmonize the balance between inflammation and tissue repair [12]. Imbalance of M1/M2 macrophage populations is associated with different diseases [5]. Uncontrolled inflammatory response driven by macrophages leads to chronic inflammation and autoimmune diseases (Figure 3) [1]. Similarly, the dysregulation of anti-inflammatory response can contribute to tumour progression and metastasis (Figure 3) [8]. In addition, prolonged inflammation and continuous activation of macrophages results in chronic diseases that may lead to the development of pathological fibrosis, a process in which normal tissue is replaced by scar tissue due to excessive deposition of ECM components. In some diseases, extensive fibrosis can ultimately lead to organ failure and death [10].

The important role of macrophages on the initiation and progress of different diseases and their participation in several body functions has made that many efforts have been devoted during the last years to design nanomaterials to target and treat macrophages [10]. Different types of biomaterials such as nanoparticles (NPs) and hydrogels are being extensively developed to target macrophages. Since macrophages are professional phagocytic cells, NPs can be exploited as vehicles that naturally target macrophages. These immune cells can easily incorporate NPs via phagocytosis, macropinocytosis, or receptor-mediated endocytosis [13]. Some types of NPs can interact with macrophages to directly modify their biological functions [12]. In addition, NPs can be used as drug delivery systems to treat macrophages involved in different diseases [2]. Several therapeutic options using functionalized NPs are being explored and developed to modulate macrophages, including macrophage depletion and phenotype repolarization (M1 to M2 or M2 to M1) depending on the phenotypic characteristics and involvement of these immune cells in each pathological process.

NPs are nanomaterials with three external nanoscale dimensions and an average size ranging from 1 to 1000 nm. Due to unique physicochemical characteristics including their size range, hydrophilic properties, and charge characteristics, NPs are widely used in the biomedical field as carriers for antigens, imaging agents, and therapeutic drugs for diagnosis or therapy [5,7]. There are several unique features that support the use of NPs for drug delivery. Most NPs are nowadays synthesized from biocompatible biomaterials that present low toxicity and can efficiently encapsulate drugs. They can penetrate physiological barriers and are stable in the bloodstream [11]. NPs can be tuned with optimal sizes, shapes, and surface modifications to improve their biodistribution, solubility, degradation, immune system evasion, and to increase circulation time and prolonged drug presence at the target tissue [11,12]. NPs can be also modified by the addition of ligands to achieve specific tissue targeting. Stimulus-responsive NPs can be also designed to achieve controlled cargo release for obtaining a precise dosage [14].

The colloidal properties of NPs can be optimized to influence their performance in macrophage-targeted therapies [15]. Most NPs are uptaken by macrophages via endocytosis [15]. During this process, NPs are retained in the endolysomal compartment in which they are degraded. Therefore, NPs need to incorporate cationic ligands or surfactants such as polyethylenimine (PEI) to destabilize the lysosomal membrane to escape [16]. NPs are internalized in a size-dependent manner [17]. Increasing the particle size improves the macrophage targeting efficiency [4]. Shape, surface moieties, and charge dictate NP binding affinity to macrophages. Macrophages expose negatively charged sialic acids on their surface promoting the phagocytosis of positively charged NPs [15]. Moreover, the addition of specific macrophage ligands to NPs can improve the attachment to receptors expressed in the macrophage surface such as mannose receptor, scavenger receptors, Fc receptors, and folate receptors to facilitate endocytosis [2,15].

This review summarizes the use of NPs to modulate macrophages involved in the initiation and progress of different diseases. We will particularly focus on NPs to target and treat macrophages involved in diseases characterized by chronic inflammation and in tumor-associated macrophages (TAMs).

## 2. NPs to Modulate Macrophages in Chronic Inflammation

Macrophage signals during injury are programmed to contain and destroy the origin of damage. The persistence of the harmful agent and the propagation of the inflammatory response leads to the imbalance between inflammatory and anti-inflammatory signals. In this scenario, macrophages are potential targets to treat inflammatory disorders such as rheumatoid arthritis, atherosclerosis, and inflammatory bowel disease. Drug-loaded NPs can be used to modulate the immunological activity of the pro-inflammatory M1 macrophages or to switch their phenotype from M1 to M2 [11,18]. These therapeutic strategies are designed to shift the dynamic balance from pro- to anti-inflammatory signals to mitigate the pathological process [5]. NP-based strategies to modulate macrophages in chronic inflammation are summarized in Table 1.

### 2.1. NPs to Induce Anti-Inflammatory Macrophage Switch

The activity of M1 macrophages is strongly associated with the progression of chronic inflammation. Some therapeutic approaches have focused on treating chronic inflammation by delivering plasmid DNA of anti-inflammatory cytokines to macrophages to induce their repolarization from M1 to M2 phenotype. It has been reported that plasmid DNA (pDNA) expressing IL-4 or IL-10 encapsulated in hyaluronic acid-polyethyleneimine (HA-PEI) NPs can modulate macrophage reprogramming to M2 [16]. HA-PEI NPs were chosen as effective vehicles for pDNA delivery to macrophages because HA targets macrophage CD44 surface marker and PEI is a positive charged polymer that improves cytosolic pDNA delivery facilitating endosomal escape via the “proton sponge effect” [16]. Internalized HA-PEI/pDNA NPs induced expression of IL-4 and IL-10 in J774 macrophages, thus leading to changes in the local environment and the subsequent repolarization of macrophages from M1 to anti-inflammatory M2a and M2c phenotypes both in vitro and in vivo in mouse peritoneal macrophages [16]. The phenotype conversion from M1 to M2 has also been achieved by the induction of the CD163 gene (a M2 macrophage marker) using mannose-PEI NPs [39]. This phenotype conversion leads to the release of anti-inflammatory cytokines and the resolution of inflammation, preventing disease progression. Similar results have also been obtained using specific inflammation-related microRNAs. MicroRNAs are small and non-coding RNAs that regulate the expression of genes at the post-transcriptional level via inhibition of translation or inducing mRNA degradation [40]. One of these microRNA, miR-223, has been reported as a potent regulator of inflammatory responses because it shifts peritoneal macrophages from M1 to M2 phenotype when they are encapsulated into HA-PEI nanoparticles [41].

### 2.2. NPs Modulating Macrophages in Rheumatoid Arthritis

Macrophage reprogramming has also been used to treat autoimmune diseases. Rheumatoid Arthritis (RA) is an autoimmune disease characterized by stiffness, pain, and swelling of several joints [42]. Macrophages play a major role in synovium inflammation. Jain et al. demonstrated that the injection of non-condensing alginate-based NPs encapsulating pDNA coding for IL-10 and modified with a tuftsin peptide (to target macrophages) was therapeutically effective treating RA in arthritic rats [19]. M1 macrophages were successfully reprogrammed by these NPs to M2 macrophages preventing the progression of inflammation and joint damage. Other authors have also achieved this macrophage polarization in RA. They have used manganese ferrite and ceria-anchored mesoporous silica NPs [20]. These nanostructures synergistically scavenged ROS and generated oxygen in environments enriched in H_2_O_2_. The injection of these NPs to rats with arthritis reduced inflammation inducing the phenotypic conversion of macrophages from M1 to M2.

Other strategies have been designed to target the synthesis of TNFα and IL-1 (both main pro-inflammatory cytokines involved in RA pathogenesis). NP-mediated TNFα knockdown in macrophages has been reported as an effective treatment for arthritis in murine models [43]. Chitosan/small interference RNA (siRNA) NPs have been used to down-regulate TNFα expression to promote systemic anti-inflammatory effects [21]. Down-regulation of TNFα and IL-1 has also been achieved by folate–Chitosan DNA NPs containing an Interleukin-1 Receptor Antagonist [22]. These NPs demonstrated a precise activity blocking the effects of IL-1 by interfering with its binding to IL-1R, thus ameliorating joint damage in RA experimental models.

Folate receptors (FR) have also been targeted to treat RA [44]. It has been reported that the expression of FRβ is abundant on activated macrophages and plays an important role on RA [45]. The folate antagonist methotrexate (MTX) has been the primary drug used to treat RA for many years. MTX inhibits DNA formation blocking the biosynthesis of nucleotides. This results in a reduced cell proliferation and the induction of apoptosis [23]. Some groups have used NPs targeting FR to deliver MTX. Thomas and co-workers have designed folic acid (FA)-conjugated G5 dendrimers to target macrophages in vitro to deliver MTX in a collagen-induced arthritis (CIA) murine model [23]. Zhao and colleagues have developed FRβ-targeting pH-responsive nanocarriers loaded with MTX in rat model of adjuvant-induced arthritis [24]. These nanocarriers responded to an acidic pH delivering the drug efficiently and reducing the progression of RA.

Other authors have used polymeric NPs that combine the anti-inflammatory drugs dexamethasone and naproxen [25]. These drugs decreased inflammation and prevented the expression of IL-12 in RAW264.7 murine macrophages. The reduction of macrophage-released IL-12 prevented the pro-inflammatory immune response and induced M1 to M2 polarization. Other authors have delivered dexamethasone to inflamed joints of CIA mice using FA-modified ROS-responsive NPs [26]. These NPs released dexamethasone and ameliorated RA interfering with the ‘iRhom2-TNF-α-BAFF’ signaling pathway.

### 2.3. NPs Modulating Macrophages in Inflammatory Bowel Disease (IBD)

Inflammatory bowel disease (IBD) is characterized by chronic inflammation of the gastrointestinal (GI) tract. There are two types of IBD: Crohn disease (CD) and ulcerative colitis (UC) [12]. Macrophages are an important source of proinflammatory cytokines (such as TNFα) that play an important role on the pathogenesis of IBD. TNFα has become an attractive target for IBD therapy using NPs. Most studies have investigated the use of different NPs containing TNFα siRNA as an interesting therapeutic strategy. Xiao and colleagues have used mannosylated NPs as delivery vehicles for TNFα siRNA [27]. TNFα siRNA demonstrated an effective activity to drastically reduce the TNFα expression and promoted anti-inflammatory effects in vitro and ex vivo leading to colitis attenuation in a dextran sodium sulfate (DSS)-induced colitis mouse model [27]. Similar results were obtained by H. Laroui and colleagues. They designed NPs with a Fab’ portion of the F4/80 antibody against murine macrophages, which also contained TNFα-siRNA showing high efficiency in the attenuation of colitis [28]. Wilson and colleagues have also encapsulated TNFα siRNA in NPs, but they have designed stimulus-responsive NPs. They have developed thioketal NPs (TKNs) that are reactive to high concentration of ROS. TKNs degradation was stimulated by tissue inflammation (where ROS concentration was high) leading to the release of the encapsulated siRNA [29]. TKNs loaded with TNFα siRNA resulted in a successful treatment for intestinal inflammation in a murine colitis model. Indeed, TNFα siRNA has also been encapsulated in galactosylated chitosan (GC) poly (lactic-co-glycolic acid) (PLGA) NPs for the treatment of experimental ulcerative colitis [30]. Other strategies have combined the delivery of TNFα siRNA with siRNA against Cyclin D1 [31]. Cyclin D1 is a cell cycle regulator that modulates cell proliferation. Overexpression of Cyclin D1 has been found in immune cells during inflammatory bowel diseases [46]. Kriegel and Amiji have used NPs in microsphere oral systems for dual siRNA (TNFα and Cyclin D1) delivery [31]. This dual treatment has shown to be more effective than each agent separately for treating IBD in a DSS-induced mouse model. Aouadi et al. developed b1,3-D-glucan-encapsulated siRNA NPs as oral delivery vehicles of Mitogen-activated protein kinase 4 (Map4k4) siRNA [32]. Map4k4 is a not well understood mediator of pro-inflammatory cytokine expression in macrophages. These authors reported that NPs releasing Map4k4 siRNA ameliorated colitis in mice via inhibition of the LPS-induced expression of TNFα and IL-1.

### 2.4. NPs Modulating Macrophages in Atherosclerosis

**Figure 4:**
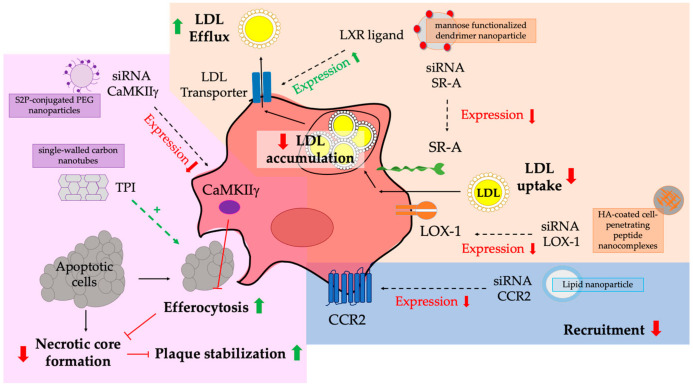
NPs to target and modulate macrophages to treat atherosclerosis. Expected therapeutic effects of functionalized NPs to fight atherosclerosis: (1) LDL uptake must be reduced, and the LDL efflux must be increased to reduce LDL accumulation (orange); (2) Macrophage recruitment must be blocked (blue); (3) Efferocytosis must be activated to prevent the formation of a necrotic core (violet). This figure shows nanoparticles-based strategies to target and treat macrophages for the treatment of atherosclerosis. LDLs: low-density lipoproteins; LXR: liver-x-receptor; siRNA: small interference RNA; SR: scavenger receptor; LOX-1: LDLs receptor 1; HA: Hyaluronic acid; CCR2: chemokine receptor 2; CaMKIIγ: Ca^2+^/calmodulin-dependent protein kinase γ; TPI: tyrosine phosphatase inhibitor 1.

Numerous potential therapies based on NPs targeting macrophages have been developed to treat atherosclerosis (Figure 4). Atherosclerosis is a chronic inflammatory disease characterized by the narrowing and thickening of the arteries caused by the build-up of plaques containing low-density lipoproteins (LDLs) and immune cells in the artery wall [47]. Accumulation of oxidized LDLs in the artery wall triggers the recruitment of immune cells and inflammation. Recruited monocytes from the bloodstream transform into macrophages and incorporate LDLs to form foam-like cells, which play a critical role in the occurrence and development of atherosclerosis [4]. Pro-inflammatory macrophages play an important role on plaque initiation and progression, and anti-inflammatory macrophages are responsible for plaque stabilization [48]. NPs modified with targeting ligands, such as mannose, hyaluronan [49], folate, DNA, peptides, antibodies, HDLs, and LDLs have been used to target intraplaque macrophages and improve the delivery of anti-inflammatory drugs to promote anti-atherosclerotic effects [4]. Statins are some of these anti-inflammatory drugs loaded in functionalized NPs to inhibit atherosclerotic plaque formation. High-density lipoproteins (HDLs) NPs have been designed as a possible delivery vehicle for statins to the liver [50]. Released statins upregulated LDL receptor expression in hepatocytes leading to the reduction of circulating LDLs and plaque formation.

Some strategies for the treatment of atherosclerosis have focused on reducing LDL accumulation in macrophages (Figure 4). Macrophages uptake oxidized LDLs through scavenger receptors (SRs) and release LDLs through the lipid transporters ABCA1 and ABCG1 [4]. Some authors have designed NPs to reduce the macrophage expression of SRs such as SR-A or oxidized LDLs receptor 1 (LOX-1) using siRNA in murine models [33,34]. Other authors have used mannose functionalized dendrimer NPs (mDNPs) to deliver the liver-x-receptor (LXR) ligand, which regulates the expression of cholesterol transporters [51]. The reduction of SR expression has been achieved using HA-coated cell-penetrating peptide (CPP) nanocomplexes (NCs) delivering siRNA against LOX-1 [34]. These NPs significantly reduced lipid accumulation and stimulated plaque regression. Zang and colleagues have used mDNPs to simultaneously deliver siRNA against SR-A and liver-x-receptor (LXR) ligand to atherosclerotic mice resulting in a greater decrease in cholesterol content in macrophages and plaque regression [33]. Other authors have used NPs to deliver siRNA to block the expression of chemokine receptors associated with monocyte recruitment as a potential treatment for atherosclerosis [4] (Figure 4). Leuschner et al. have designed lipid NPs as vehicles for siRNA delivery to block the chemokine receptor 2 (CCR2) expression and the subsequent monocyte recruitment in atherosclerotic mice [35]. This CCR2 downregulation has also been achieved by another group through EGFP-EGF1-conjugated PLGA nanoparticles and shRNA against CCR2 in RAW264.7 macrophages [36].

A different strategy for the treatment of atherosclerosis is the modulation of macrophages to enhance efferocytosis (Figure 4). Efferocytosis is the phagocytic clearance of dying cells and apoptotic and necrotic debris by professional phagocytes [52]. Apoptotic cells are cleared very quickly mainly by macrophages in almost all tissue beds. However, their removal appears to be significantly impaired in atherosclerotic diseased blood vessels. The therapeutic intervention to recover from this efferocytosis defect is very relevant in atherosclerosis to prevent the formation of a necrotic core that destabilizes the plaque formation [4]. Ca^2+^/calmodulin-dependent protein kinase γ (CaMKIIγ) blocks macrophage efferocytosis leading to the conversion of stabilized atherosclerotic plaques into necrotic lesions. Tao et al. have tested the efficacy of S2P-conjugated 1,2-Distearoyl-sn-glycero-3-phosphorylethanolamine (DSPE) polyethylene glycol (PEG) NPs delivering siRNA to macrophages to block the expression of CaMKIIγ and they have demonstrated that this therapy promotes plaque stabilization in mice [37]. Similar results have been obtained by Zhang et al. using a novel macrophage-selective carrier system consisting of single-walled carbon nanotubes with a small molecule enzymatic inhibitor (tyrosine phosphatase inhibitor 1) that is released in a pH-dependent manner to stimulate macrophage efferocytosis of apoptotic cell debris via the CD47-SIRPα signaling pathway [38]. 

## 3. NPs to Stimulate Tissue Repair and Regeneration

M2 macrophages are actively involved in tissue repair and wound healing. For this reason, many investigations nowadays are dedicated to design NPs to stimulate the M2 macrophage phenotype for tissue or organ regeneration. Indeed, some of these strategies involve M2 repolarization to combine an anti-inflammatory and a pro-regenerative effect [53]. Nanoparticle-based strategies to modulate macrophages for tissue repair are summarized in Table 2.

### 3.1. NPs Modulating Macrophages in Tissue Regeneration

A study carried out by Raimondo and Mooney has reported that IL-4-conjugated gold NPs (AuNPs) are efficient in enhancing regeneration of murine skeletal muscle after ischemic injury via modulation of M2 macrophage polarization [54,70]. Ge and colleagues have also shown direct effects of bare AuNPs to stimulate macrophage repolarization for murine skeletal muscle regeneration [56]. Indeed, they reported that AuNPs significantly enhanced myogenic differentiation of myoblasts promoting in vivo regeneration in models of muscle defects in rats. AuNPs have also been demonstrated to efficiently stimulate regeneration of lost periodontal apparatus after periodontitis [55]. Periodontitis is a bacterially induced chronic inflammatory disease that progressively destroys the supporting structures of teeth. AuNPs induced a macrophage phenotype switch to M2. AuNPs increased bone morphogenetic protein-2 (BMP-2) expression in macrophages, leading to periodontal ligament cells differentiation and periodontal tissue regeneration in murine models.

TiO_2_ nanotubes have been reported to promote osteogenesis via crosstalk between macrophages and mesenchymal stem cells (MSCs) under oxidative stress, stimulation of M1 to M2 macrophage transition and reduction of early inflammation [56]. Mesoporous silica NPs (MSNs) have been employed to inhibit the inflammatory response and to enhance the osteogenic differentiation of bone marrow mesenchymal stromal cells (BMDMs) through their immunomodulatory effects on macrophages [57]. In addition, M. Shi et al. have incorporated copper to MSNs to achieve osteogenic differentiation via the Oncostation M pathway [58]. Bone regeneration has also been achieved by Lee and co-workers via inhibition of bone marrow-derived macrophage differentiation to osteoclast using functionalized gold nanoparticles conjugated with alendronate [59,71].

### 3.2. NPs Modulating Macrophages in Myocardial Infarct Repair

**Figure 5:**
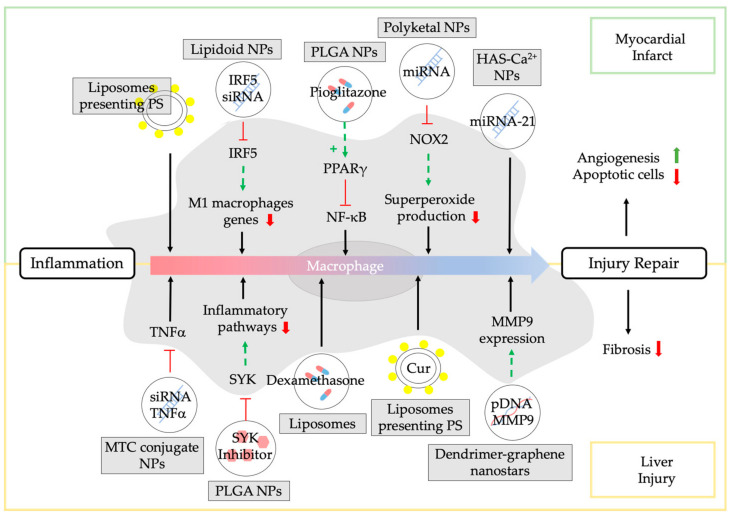
NPs to target and treat macrophages for tissue repair. The figure summarizes different therapeutic approaches to design NPs loaded with different biomolecules to stimulate tissue repair after liver injury (yellow rectangle) and myocardial infarct (green rectangle). All these therapies focus on reducing inflammation and/or inducing a pro-regenerative macrophage phenotype to promote repair. PS: phosphatidylserine; NPs: Nanoparticles; IRF5: interferon regulatory factor 5; SiRNA: small interference ribonucleic acid; PLGA: poly (lactic-co-glycolic acid); PPARγ: peroxisome proliferator-activated receptor-gamma; NF-κB: nuclear factor kappa light chain enhancer of activated B cells; Nox2: NADPH oxidase 2; HAS: hyaluronan-sulfate; TNFα: tumor necrosis factor alpha; MTC: mannose-modified trimethyl chitosan-cysteine; SYK: Spleen Tyrosine kinase; Cur: Curcumin; pDNA: plasmid DNA; MMP9: matrix metalloproteinase 9.

Unstable atherosclerotic plaques are prone to rupture, leading to thrombosis, myocardial infarction, or stroke [3]. Myocardial infarction (MI) is the result of partial or complete coronary artery occlusion that leads to blood flow reduction. Prolonged myocardial ischemia stimulates cardiomyocyte death. The infarcted myocardium heals through the formation of a non-contractile scar tissue [12,61]. The appropriate myocardial healing is guided by macrophages [4]. For this reason, numerous NPs have been developed to target macrophages and achieve myocardial infarct repair (Figure 5).

Harel-Adar et al. have investigated intravenous administration of phosphatidylserine (PS)-presenting liposomes as a new strategy to achieve myocardial infarct repair in a rat model of acute MI [60]. These authors designed liposomes with PS to mimic apoptotic cells and to induce macrophage transition and myocardial infarct repair. Cardiac healing has also been investigated by Bejerano and colleagues. They delivered miRNA-21 encapsulated into hyaluronan-sulfate (HAS)-Ca^2+^ NPs to cardiac M1 macrophages at the infarct zone inducing their repolarization to an anti-inflammatory and reparative M2 phenotype [61]. miRNA-21 upregulation increased angiogenesis, reduced the number of apoptotic cells, and improved cardiac healing in mice by promoting inflammation resolution. The same results have been obtained in another study using miRNA NPs [62]. These authors used miRNA polyketal NPs to downregulate the expression of NADPH oxidase 2 in mice, a protein responsible of superoxide production, an important mediator in inflammation.

Courties et al. incorporated siRNA targeting interferon regulatory factor 5 (transcription factor that up-regulates genes associated with M1 macrophages) into lipidoid NPs to achieve attenuation of M1 macrophage polarization, inflammation resolution, and tissue regeneration in a mouse model of MI [63]. M. Tokutome et al. achieved M2 polarization and subsequent cardiac healing in animal models encapsulating pioglitazone in PLGA NPs, activating the macrophage peroxisome proliferator-activated receptor-gamma (PPARγ) [64]. PPARγ is a nuclear receptor that can repress nuclear factor kappa light chain enhancer of activated B cells (NF-κB), the master regulator of inflammatory responses.

### 3.3. NPs Modulating Macrophages in Chronic Liver Injury

During the past decades, it has been demonstrated that hepatic macrophages hold central functions in the initiation, propagation, and perpetuation of inflammation in liver injury [72]. Different strategies have been suggested to treat liver inflammation and to target hepatic macrophages [73,74] (Figure 5). He and co-workers have suggested the use of mannose-modified trimethyl chitosan-cysteine (MTC) conjugate NPs containing TNFα siRNA [65]. These NPs attained a significant reduction in TNFα expression, blocked liver injury progression and reduced mortality in mice with acute hepatic injury.

Other authors have described PLGA NPs with a Spleen Tyrosine kinase (SYK) pathway inhibitor to target and treat macrophages in chronic liver injury [66]. SYK is an important mediator in the downstream signaling events that drive inflammatory pathways. SYK inhibition using these NPs resulted in a significant reduction in inflammation, hepatic injury, and fibrosis in mice. Similar results have been obtained in experimental models of acute and chronic liver injury in mice using liposomes loaded with dexamethasone [67]. Dexamethasone induced anti-inflammatory M2 polarization of hepatic macrophages and reduction of liver injury and liver fibrosis.

Wang et al. have used PS-modified nanostructured lipid carriers containing curcumin [68]. PS-containing NPs are used to mimic apoptotic cells that can be specifically recognized by macrophages [75]. The use of these NPs resulted in a substantial reduction in liver damage and fibrosis in rats [68].

Other authors have focused on stimulating the selective production of collagenases to reduce liver fibrosis [69]. Dendrimer-graphene nanostars have been designed to deliver a plasmid expressing the collagenase metalloproteinase 9 into inflammatory macrophages in cirrhotic livers. These NPs efficiently degraded collagen in vitro and promoted a phenotypical transformation from inflammatory to pro-regenerative and anti-inflammatory macrophages in a mouse model of liver fibrosis caused by chronic intraperitoneal administration of CCl_4_. This targeted gene therapy reduced selectively and locally the presence of collagen fibers, decreased hepatic injury, and allowed hepatic regeneration [69].

## 4. Nanoparticles to Target and Treat Tumour-Associated Macrophages

### 4.1. Specific Differential Phenotype of Tumour-Associated Macrophages

Tumor-associated macrophages (TAMs) are the most abundant immunosuppressive cells in the tumor microenvironment. They play a fundamental role in the promotion of tumor initiation, growth, and progression [76]. In the initial phase of cancer, they activate antitumor immunity. When the tumor is firmly established, they promote angiogenesis, immunosuppression, and metastasis [77,78]. Numerous tumor-derived chemoattractants are crucial for recruiting monocytes into the tumor milieu and to promote their transition to TAM. These include chemokines such as CXCL12 (stromal cell-derived factor 1α), CCL3 (macrophage inflammatory protein (MIP) 1α), CCL2 (MCP-1) and CCL4 (MIP1β), interleukins (IL-6 and IL-1β), and cytokines (colony stimulating factor 1 (CSF-1) and VEGF A) [79,80,81,82,83].

TAMs are involved in a multitude of phenomena related to cancer growth and progression. Immunosuppression is one of the key features of TAMs as they suppress CD8+ T cell activation, a major mechanism of anti-tumor immunity [48,49]. They also participate in the molecular dysfunction of natural killer (NK) and NK T cells. TAM-derived IL-10 inhibits local IL-12, which is essential for the cytotoxic activity of NK cells [84]. TAMs secrete numerous pro-angiogenic factors such as VEGF and platelet-derived growth factor (PDGF), and chemokines such as CCL2 and CXCL9, which provide a vascular niche for the tumor. This not only maintains tumor growth through neovascularization, but also promotes the dissemination to distant organs [45,47]. TAMs are also involved in epithelial to mesenchymal transition necessary for the formation of a metastatic niche for the spread of cancer cells to distant organs [85]. Cancer cell stemness is regulated by stromal cells [83]. In this context, inflammatory signals derived from TAMs, like IL-6, facilitate the expansion of these cancer stem cells and lead to a poor response to chemotherapy [83,86]. TAMs may influence conventional cancer treatments such as chemotherapy and radiotherapy by the promotion of tumor tissue repair responses and a protective niche for cancer stem cells [76].

TAMs have been recognized as potential therapeutic targets for cancer immunotherapy. Macrophage-related therapeutic approaches are already in clinical trials, but they still need to be supplemented with conventional cancer treatments such as standard cytoreductive therapies, angiogenesis inhibitors, and check-point inhibitors immunotherapy [76].

NPs have been widely designed as drug delivery systems to treat cancer. They can prolong the retention time of chemotherapeutics and achieve targeted delivery, thus reducing toxicity. They provide the opportunity to deliver drugs directly to TAMs. The modulation of TAM phenotype could be an effective strategy in cancer immunotherapy. Engineered NPs can target TAM components and transform immunosuppressive TAMs into immunocompetent macrophages to improve the efficacy of cancer immunotherapy [87].

### 4.2. NPs to Target TAM for Cancer Diagnostics and Prognosis

The fact that TAM display a very particular phenotype can be used to design selective diagnostic strategies for some types of solid tumors and even to define their prognosis because TAM invasion into tumor stroma has been associated with a worse prognosis in several types of cancers [79]. NPs designed to target tumors are preferentially phagocytosed by macrophages as any other NPs. In this context, Leimgruber and colleagues have demonstrated that AMTA680 magnetofluorescent injectable NPs label endogenous TAM allowing the tracking of these immune cells within the microenvironment of soft tissue sarcoma, lung carcinoma, and colon adenocarcinoma [88]. AMTA680 NPs consisted of a fluorescent dye for optical imaging (VT680) that emitted in far-red wavelength and a super-paramagnetic core for MRI. AMTA680 preferentially targeted “M2-like” TAMs expressed high levels of F4/80 and VEGF [88]. Iron oxide NPs have also been employed for MRI diagnosis in tumors and to quantitatively monitor the TAM presence in breast cancer [89]. Ferumoxytol NPs (clinically approved iron NPs that are naturally engulfed by TAM) have been used for the MRI diagnosis of anaplastic thyroid cancer [90] and melanoma [91]. This type of aggressive cancer displays high TAM infiltration, which can be monitored by MRI with NPs targeting macrophages [90]. Surface modification of iron oxide NPs with folate has demonstrated to increase the uptake in macrophages via folate receptor-β, which is highly expressed in TAM [92]. Perfluorocarbon nanoemulsions have also been employed for murine breast cancer MRI diagnosis [93]. Pérez-Medina et al. have developed an ^89^Zr-modified reconstituted HDL (rHDL) nanotracers for TAM PET imaging in a murine model of breast cancer [94]. These nanotracers accumulated in TAM-rich tumor areas. The rHDL were prepared by mixing dimyristoylphosphatidylcholine (DMPC) vesicles with apoA-I resulting in discoidal NPs [94]. ^64^Cu loading in NPs has been investigated for PET imaging [95]. Kim et al. have demonstrated that ^64^Cu-labeled polyglucose NPs were effective for PET breast cancer diagnosis. In another study, Locke et al. used mannosylated liposomes loaded with ^64^Cu for PET pulmonary adenocarcinoma diagnosis [96]. Histological methods to quantify TAM in tumors may result in being invasive. Nanoconstructs that can be tracked by MRI or PET offer the opportunity to non-invasively classify tumors with high TAM infiltration and can be applied to monitor TAM-targeted immunotherapies in clinical trials. Near-infrared fluorescent silica coated iron oxide NPs have also been used for the accurate delineation of glioblastoma multiforme [97]. This type of glioblastoma has abundant infiltration of macrophages at the tumor margins. Fluorescent labeling of TAM served for its complete surgical resection. Indeed, these authors demonstrated that these NPs cross the blood–brain barrier [97]. This and other diagnostic and therapeutic strategies are summarized in Table 3.

### 4.3. NPs to Inhibit Macrophage Recruitment and to Deplete TAM in Tumors

**Figure 6:**
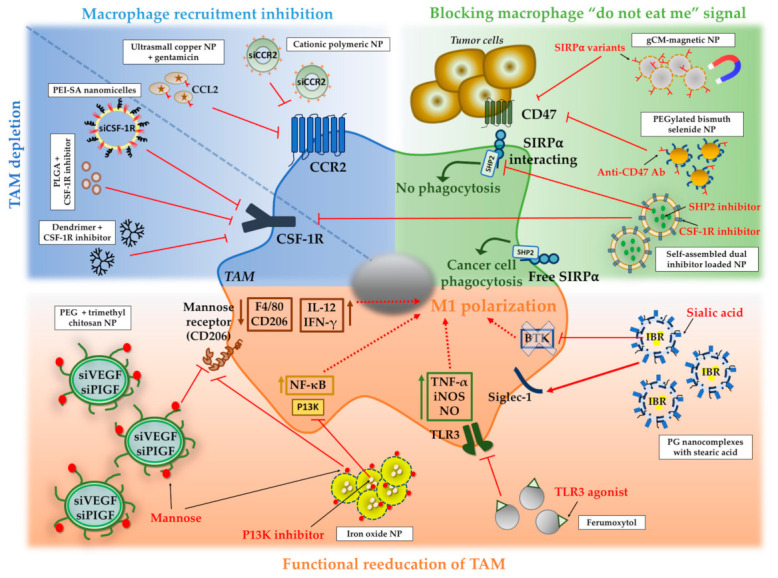
Strategies for the targeting of TAM using NPs. Four main strategies have been described for TAM-targeted therapies: (1) inhibition of macrophage recruitment to the tumor (blue); (2) TAM depletion in the tumor microenvironment (blue); (3) blockade of macrophage immune checkpoint (“do not eat me” signal) (green); and (4) functional re-education of TAM to a M1 phenotype (orange). The image illustrates different molecular targets and different types of NPs that have been described to treat TAM. NP: nanoparticles; PEI-SA: polyethylenimine and stearic acid; PLGA: poly (lactic-co-glycolic acid); CSF-1R: Colony stimulating factor 1 receptor; TAM: tumor-associated macrophages; CCR2: C-C Motif Chemokine Receptor 2; SHP2: Src homology region 2 phosphatases; SIPRα: macrophage signal-regulatory protein alpha; Ab: antibody; gCM: genetically engineered cell membrane; PEG: polyethylene glycol; VEGF: vascular endothelial growth factor; PIGF: placental growth factor; IFNγ: interferon γ; NF-κB: nuclear factor kappa-light-chain-enhancer of activated B cells; P13K: Phosphoinositide 3-kinases; TNF-α: tumor necrosis factor; iNOS: nitric oxide synthases; NO: nitric oxide; TLR: toll-like receptor; IBR: Ibrutinib; BTK: Bruton’s tyrosine kinase.

In recent years, different strategies have been suggested for TAM-targeted therapies. In the first stages of cancer, many efforts have been focused on designing NPs to prevent macrophage recruitment to the tumor area (Figure 6). Cancer cells secret a myriad of chemoattractant molecules to recruit circulating monocytes from the blood stream. The inhibition of pivotal signaling pathways in monocyte recruitment, such as CCL2/CCR2, has been investigated to design NPs that block monocyte accumulation in tumors and transformation to TAM [87,98,99]. Shen et al. designed cationic polymeric NPS shaped with copolymer poly(ethylene glycol)-block-polylactide (PEG5K-b-PLA11K) and a cationic lipid (*N*,*N*-bis(2-hydroxyethyl)-*N*-methyl-*N*-(2-cholesteryloxycarbonyl aminoethyl) ammonium bromide) that were engulfed by circulating Ly6C^hi^ monocytes [98]. These NPs encapsulated a siRNA that blocked CCR2 receptor expression preventing TAM formation in an orthotopic murine model of breast cancer [98]. In a different study, ultrasmall copper NPs were designed to target CCR2 with an antibody [99]. These NPs encapsulated a chemotherapeutic agent resulting in a targeted therapy for pancreatic ductal adenocarcinoma with reduced toxicity.

Depletion of TAM has also been reported as a potential strategy to treat the tumor microenvironment and to prevent tumor progression and dissemination (Figure 6). Colony stimulating factor 1 receptor (CSF-1R) controls the function, differentiation, and the formation of macrophages and it is overexpressed in TAMs. Cancer cells secrete CSF-1 to activate CSF-1/CSF-1R pathway and TAM formation. NPs targeting CSF-1R have been suggested as a possible therapy for TAM depletion. For example, a composition of polyethylenimine and stearic acid (PEI-SA) nanomicelles containing a siRNA against CSF-1R has been used to block CSF-1 receptor [100]. Qian et al. have also used NPs containing a siRNA against CSF-1R [101]. These NPs consisted of 1,2-dimyristoyl-sn-glycero-3-phosphocholine (DMPC) and cholesterol oleate. TAM targeting was carried out with a peptide with high specificity for M2 macrophages (M2pep) [101]. This peptide selectively targeted TAMs without targeting other similar cells such as alveolar macrophages [118]. The use of NPs loaded with CSF-1R inhibitors, such as BLZP945 and PLX3397, has also been suggested by several authors [102,103,104,105]. PLGA NPs loaded with a CSF-1/CSF-1R inhibitor (PLX3397) have been designed to deplete TAM in B16F10 tumors, a melanoma experimental model [102]. Cuccarese and colleagues have designed fluorescent injectable NPs loaded with the same PLX3397 CSF-1R inhibitor to treat murine pulmonary carcinoma [105]. Both types of NPs loaded with CSF-1R inhibitors reduced “M2-like” TAM number. NPs targeting CSF-1 in TAM have also demonstrated improved response to therapy in glioblastoma [103]. This study was carried out using hydroxyl dendrimer NPs with BLZ945 as CSF-1R inhibitor [103]. These NPs allowed sustained intracellular and intratumoral drug release [103]. This therapeutic approach resulted in “M2-like” TAM depletion (a decrease in arginase levels) and intratumoral accumulation of cytotoxic T cells indicating an increase in antitumor immunological activity. Moreover, survival rate improved in mice treated with these NPs compared with the group treated with free drug [103]. Dextran-grafted poly(histidine) copolymer NPs have also been used to target TAM and release BLZ945 CSF-1R inhibitor in a breast cancer experimental model [104]. Therefore, the use of NPs targeting TAM in aggressive tumors (such as glioblastoma or melanoma) may be a promising approach to improve immunological therapies and to reduce the side effects caused by conventional cytotoxic therapies.

### 4.4. NPs to Block the Macrophage “Do Not Eat Me” Signal

Cancer cells develop diverse mechanisms to escape macrophage phagocytosis, thus promoting an immunosuppressive tumor microenvironment. The overexpression of membrane CD47 (also known as integrin associated protein) is a potent “do not eat me” signal for macrophages and allows cancer cells to evade macrophage phagocytic activity [119,120]. The crosstalk between the cancer cell CD47 and the macrophage signal-regulatory protein alpha (SIRPα) has been designated as the innate immune checkpoint [119]. CD47/SIRPα crosstalk has been conceptually chosen to develop NPs that could efficiently block cancer resistance to macrophage-mediated phagocytosis (Figure 6).

Rao and colleges have constructed genetically engineered cell-membrane (gCM)-coated magnetic NPs overexpressing SIRPα variants with high efficiency for CD47 [107]. This allowed the blockade of CD47/SIRPα crosstalk under magnetic activation in murine models of melanoma and triple negative breast cancer. This therapy prolonged mouse survival, controlled tumor growth, promoted cancer cell cytophagy, boosted T-cell mediated devastation of cancer cells, and improved antigen presentation by macrophages and dendritic cells. These constructs reduced immune clearance of magnetic NPs, demonstrating a promising capacity of biomimetic nanomaterials for cancer immunotherapy [107]. CD47-targeted bismuth selenide (Bi2Se3) NPs have also been employed to deplete TAMs in tumors and to promote macrophage-mediated phagocytosis of cancer cells [108]. These NPs presented an excellent photothermal performance and efficiently increased the temperature of the tumor. This photothermal therapy reduced tumor size in 4T1 tumor-bearing mice, a breast cancer model. A further modification of Bi2Se3 NPs consisted of polyethylene glycol (PEG) functionalization and an anti-CD47 antibody (Ab) coating to target cancer cells [108]. Ab-PEG-Bi2Se3 also were useful for IR thermal imaging, which helped to track the location of the agent in cancer cells [108].

Another strategy to inhibit the innate immune checkpoint is blocking the molecules that are downstream of SIRPα. Src homology region 2 phosphatase (SHP2) is a downstream effector responsible for SIRPα function in neurons, dendritic cells, and macrophages [120]. Some authors have designed self-assembled dual inhibitor-loaded NPs to target “M2-like” TAM [106]. These NPs consisted of different self-assembled colipids: 1,2-Distearoyl-sn-glycero-3-phosphorylethanolamine (DSPE), PEG, and Phosphatidylcholine (PC). These constructs have been used for the delivery of two key TAM-related pathway inhibitors, SHP2 and CSF-1R inhibitors. NPs with both inhibitors increased macrophage phagocyte ability as compared to free drugs [106]. Simultaneous and sustained co-release of different drugs can be challenging, but NPs provide the opportunity to transport and release more than one drug safely and effectively. Therefore, we must consider NPs as versatile platforms useful for the simultaneous administration of different combinations of immunotherapies.

### 4.5. NPs to Switch TAM to an Antitumor “M1-Like” Phenotype

Macrophage activity is molded by cancer cells to help tumor growth. However, macrophages are very plastic cells that may be re-educated by functionalized NPs to switch from “M2-like” TAMs to an M1 pro-inflammatory phenotype to detect and destroy cancer cells (Figure 6) [78,121,122].

M1 polarized macrophages help to combat tumor growth via activation of phagocytosis and T-cell mediated immune response. The differential phenotype of TAMs is still being investigated to find therapeutic targets to re-polarize these macrophages to an “M1-like” phenotype [76,77,78]. For example, VEGF and Placental growth factor (PIGF) are overexpressed in “M2-like” TAM. The reduction of these growth factors leads to the polarization of macrophages towards a M1 phenotype and avoids tumor neovascularization [79,81]. Song et al. designed NPs with PEG and trimethyl chitosan modified with mannose [109]. They included two siRNAs to block the expression of VEGF and PIGF in TAMs. Mannose modification allowed NPs to efficiently target M2 macrophages, as its receptor is highly expressed in TAMs. They used two siRNAs (siVEGF and siPIGF) to maximize anti-tumor efficacy in a breast cancer model. They found lower colocalization of F4/80 and CD206 (mannose receptor) due to the treatment with these NPs, indicating a decrease in the M2 phenotype. In contrast, IL-12 and IFN-γ cytokine levels increased, resulting in a polarization towards the M1 phenotype (involving anti-tumor immunological activity and the activation of phagocytosis of cancer cells by macrophages) [109]. Gold NPs decorated with thiolated-PEG-COOH polymer, M2pep to target TAM, and thiolated anti-VEGF siRNA have also been developed for the induction of long lasting gene therapy and TAM M1 polarization via VEGF silencing [110].

It has been described that some microRNAs, such as miR-125B, are involved in the repolarization of macrophages from a pro-tumoral M2 phenotype to an antitumor M1 phenotype [123]. In this context, Parayath and colleagues developed CD44-targeted hyaluronic acid-poly (ethylenimine) (HA-PEI)-based NPs loaded with miR-125b to treat non-small cell lung cancer in mice [111]. These NPs promoted an increase in the synthesis of iNOS and a decrease in arginase-1 promoting M1 macrophage polarization [111]. Other authors have used anionic magnetic NPs to transfect the same miR-125b and also observed M1 macrophage polarization in an orthotopic mouse model of breast cancer [112].

Iron oxide NPs have also been employed for TAM re-education as they display intrinsic ability to promote M1 polarization in TAM [113,124,125,126]. In this context, ferumoxytol NPs, clinically approved iron oxide NPs, have been used to design a targeted therapy to TAM [113]. A toll-like receptor 3 (TLR3) agonist was attached to the NP surface to target TAM. Ferumoxytol displayed anti-tumor effects inducing a pro-inflammatory response in macrophages. Its combination with TLR3 agonist increased macrophage polarization to a M1 phenotype in a melanoma cancer model [113]. These NPs efficiently increased TNF-α, iNOS levels, and NO secretion, inducing a pro-inflammatory response in macrophages and the activation of T-cell immunity. The therapy with these NPs induced regression in primary melanoma and in lung metastases [113].

The direct induction of the M1 phenotype activating key inflammatory signaling pathways by functionalized NPs has also been explored [127]. NF-κB is a family of highly conserved transcription factors and a master regulator of inflammatory responses in macrophages [128]. In recent years, numerous investigations have arisen using modified NPs to transform TAM into M1 macrophages via induction of NF-κB-related inflammatory pathways. For example, iron oxide NPs loading a P13K inhibitor, 3-methyladenine (3-MA), modified with mannose have been used for functional TAM reeducation [114]. The transcription factor NF-κB successfully activated TAM to induce M1 polarization [114]. Iron oxide NPs coated with biomimetic membranes from LPS-treated macrophages have also been designed and tested for TAM re-education in a murine model of breast cancer [115]. These NPs were decorated with a TLR7 agonist to boost the activation of NF-κB [115]. Other authors have also suggested the use of TLR7 and TLR8 agonists to induce the activation of NF-κB to enhance immunotherapy efficacy and to increase TNF-α and IL-6 secretion [129]. For example, Shan et al. used human ferritin heavy chain nanocages modified with M2pep to deliver a TLR agonist in a murine breast cancer model [116]. TLR agonist triggered TLR signaling and polarized TAM into M1 inflammatory macrophages [116].

Bruton’s tyrosine kinase (BTK) is a non-receptor kinase belonging to the Tec family of kinases and has been found overexpressed in TAM [130]. This signaling pathway has recently been suggested as an interesting therapeutic target for myeloid cells in the tumor microenvironment and to re-educate TAMs [130]. Qiu and colleagues developed NPs with phosphatidylglycerol nanocomplexes conjugated and decorated with stearic acid [117]. They have encapsulated a small molecule BTK inhibitor, Ibrutinib (IBR), for targeted immunotherapy. They also included sialic acid linked to Sigle-1 cell adhesion molecule (overexpressed in TAM) on the NPs’ surface to target TAM. The inhibition of BTK in TAMs resulted in a reduced release of TH_2_ tumorigenic cytokines and impaired angiogenesis. The nanoconstructs efficiently suppressed tumor growth and polarized TAM to a M1 phenotype [117].

## 5. Nanocomposite Hydrogels to Modulate Macrophages

Hydrogels are three-dimensional cross-linked polymer networks that contain high amounts of water [131]. Hydrogels are drug delivery vehicles that can be used as site-specific drug-controlled release systems. The main characteristics of hydrogels are protection, targeting, and local and controlled drug delivery by swelling and shrinkage [132]. Enzymatic, hydrolytic, or environmental stimuli trigger hydrogel drug release at the implantation site [132]. The high-water content provides physical similarities to natural tissue ECM and excellent biocompatibility. The crosslinked network prevents the access of enzymes, thus protecting the drug from premature degradation [131].

Hydrogels are also widely used in the field of tissue engineering and regenerative medicine. Their structure, which mimics native tissue, can provide an ideal environment for cell survival. Heterogeneous combinations of hydrogels, cells, NPs, and bioactive molecules are state-of-the-art technologies that play a major role on the field of tissue regeneration nowadays [133].

**Figure 7:**
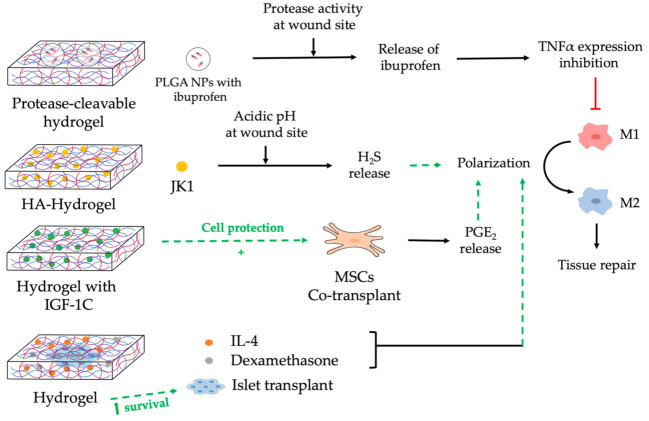
Nanocomposite hydrogels to modulate macrophages. This figure illustrates four examples of hydrogels designed to induce macrophages polarization for tissue repair. The first hydrogel encapsulates NPs with anti-inflammatory drugs that are released upon exposure to protease activity. The second hydrogel contains a donor (JK1) that can be triggered in a pH-dependent manner to release pro-regenerative molecules (H_2_S). The third hydrogel is loaded with molecules (IGF-1C) that promote co-transplanted MSCs survival, which modulates macrophage function. The fourth hydrogel encapsulates transplanted cells (islet transplant) and pro-regenerative molecules (IL-4, Dexamethasone). PLGA: poly (lactic-co-glycolic acid); NPs: nanoparticles; TNFα: tumor necrotic factor alpha; HA: hyaluronan; H_2_S: hydrogen sulphide; Insulin-like growth factor 1 (IGF-1C); MSC: mesenchymal stem cells; PGE2: prostaglandin E2; IL: Interleukin.

Hydrogels have also been used as modulators of the immune system by encapsulating: (1) immunomodulatory components, (2) anti-inflammatory or pro-regenerative bioactive molecules, and (3) cells that can interact or modulate immune cells [12]. Namely, we will focus on hydrogels used to modulate macrophages. J. Chen and colleagues synthesized a system of double hydrogel layers on titania nanotubes [134]. They introduced IFN-*γ* between the double hydrogel layers and IL-4 was encapsulated into the titania nanotubes. This dual system allowed the initial release of IFN-*γ*, which stimulated the switch of macrophages to M1 in three days and the subsequent release of IL-4 at day 4, which displayed anti-inflammatory effects mediated by the macrophage switch to M2 macrophage phenotype [134]. This is a demonstration that hydrogels can encapsulate different types of cytokines to achieve differential functions. Hydrogels loading anti-inflammatory drugs have also been designed to modulate macrophages. For example, protease-cleavable hydrogel has been designed to locally deliver PLGA NPs loaded with ibuprofen to inflamed tissues where elevated protease activity is found [135]. Delivered ibuprofen inhibited TNFα expression in RAW 264.7 murine macrophages (Figure 7).

Nanocomposite hydrogels have also been used to modulate macrophages to boost wound repair. M. Xian and collaborators have designed hydrogen sulphide (H_2_S) donors called JK1 that release H_2_S in a pH-dependent manner [136]. H_2_S is known to have an important role in wound repair due to its anti-inflammatory activity through the conversion of macrophages to a M2 phenotype [137]. It has been demonstrated that the acidic pH in wounds during the inflammatory stage triggers the release of H_2_S from JK-donors [136]. J. Wu and coworkers designed HA-hydrogels as delivery systems for JK1 and they achieved a pH-dependent prolonged H_2_S releasing profile [138]. HA-JK1 hydrogel promoted the conversion to M2 macrophages that contributed to wound healing in mice (Figure 7). Other authors have also achieved M2 polarization and wound healing in murine models, but by using adhesive hydrogels containing miR-223 HA-PEG NPs [139]. Another immunomodulatory approach to induce wound repair was described by Lohmann et al. [140]. They designed a glycosaminoglycan-based hydrogel that entrapped inflammatory chemokines, such as MCP-1(monocyte chemoattractant protein–1), IL-8 (interleukin-8), MIP-1α (macrophage inflammatory protein–1α), and MIP-1β (macrophage inflammatory protein-1β) for monocyte recruitment. This strategy successfully rescued defective wound healing in mice overcoming their limited capacity of inflammation resolution.

Hydrogels have also been used for tissue and organ regeneration. MSCs transplant has been considered a good treatment for several immune disorders because they are known to secrete prostaglandin E_2_ (PGE_2_), which promotes M2 macrophage polarization and tissue repair [141]. MSCs have been used in chitosan-based injectable hydrogels combined with entrapped C domain peptide of insulin-like growth factor 1 (IGF-1C) for colitis in mice [142]. Hydrogels containing growth factors mimic native stem cell microenvironments [143]. Therefore, these authors provided a supportive niche for transplanted stem cells that facilitated their survival and enhanced their therapeutic effects. Overall, IGF-1C hydrogels protected MSCs, which released high levels of PGE_2_ in an experimental mouse model of colitis [142]. PGE_2_ released from MSCs in these hydrogels induced the M2 macrophage phenotype reducing inflammation and promoting colon regeneration (Figure 7). Double network hydrogel based on hyaluronic acid and squid cartilage type II gelatin were synthesized for costal cartilage defect reconstruction [144]. This hydrogel effectively induced M2 polarization and promoted the subsequent chondrogenesis.

NPs in silk hydrogels to modulate macrophages are also a promising treatment for type 1 diabetes. Kumar and colleagues designed hydrogels containing IL-4 and Dexamethasone to encapsulate pancreatic islets [145]. The structure of these hydrogels allowed the preservation of islet function and viability. The release of IL-4 and Dexamethasone from these hydrogels modulated macrophage phenotype to stimulate tissue regeneration in mice (Figure 7).

## 6. Conclusions and Future Perspectives

This review has summarized the use of NPs to modulate the immunological function of macrophages involved in disease with special emphasis on chronic inflammation, tissue regeneration, and cancer.

NPs are widely used as drug or gene delivery carriers to target and treat macrophages. NPs to treat macrophages in chronic inflammation have been designed either to reduce the pro-inflammatory activity of M1 macrophages or to induce an anti-inflammatory M2 phenotype. Hydrogels with entrapped NPs have been investigated to promote macrophage M2 conversion to induce tissue regeneration. NPs to treat TAMs in cancer have been designed to inhibit TAM recruitment or accumulation, or to re-educate TAMs to restore functional phagocytosis and M1 phenotype.

The design of NPs to target and treat macrophages in chronic inflammation is mainly focused on the modulation of pro-inflammatory or anti-inflammatory cytokines or signaling pathways. A promising target for the treatment of inflammatory bowel disease is the interference of TNFα expression using thioketal NPs or other functionalized NPs. pH-responsive nanocarriers loaded with methotrexate are some of the most interesting strategies to target and treat macrophages in rheumatoid arthritis. Therapeutic strategies using NPs for atherosclerosis have focused on reducing LDL accumulation in macrophages or macrophage recruitment or enhancing efferocytosis. NPs designed to reduce macrophage LDL accumulation are excellent options to decrease macrophage cholesterol content and plaque regression. NPs stimulating M2 phenotype repolarization are some of the most effective to achieve myocardial infarct or hepatic repair. Inorganic NPs have demonstrated successful outcomes for muscle and bone regeneration.

The design of NPs to target and treat macrophages in cancer is mainly focused on the modulation of TAM in tumors. NP-based strategies for TAM depletion and inhibition are interesting approaches with an excellent potential to be combined with other immunotherapies. The re-education of TAM using siVEGF-NPs to restore the macrophage anti-cancer immunity appears as a promising approach as well. Similarly, NPs delivering PI3K and TLR3 inhibitors to macrophages also show high effectivity to polarize macrophages towards a pro-inflammatory and antitumoral phenotype. The combination of NPs delivering re-education agents to macrophages with other anti-cancer agents is a promising approach to increase antitumor effectiveness.

Despite the potential of these therapeutic approaches, more studies are needed to further elucidate the mechanisms by which NPs are incorporated by macrophages and how this selective uptake can be used to modulate these immune cells using functionalized NPs. Macrophages are very plastic cells. We still need more precise biological data to understand the array of macrophage subtypes and their precise involvement in every stage of disease to design specific therapies. A deeper understanding of the different cell-surface markers is critical to design selective NPs for a specific macrophage subset to avoid off-target effects, as macrophages with different phenotypes may co-exist in the same area of a dysfunctional tissue. The Ockham’s concept supports that the simplest explanation is usually the best option to be tested. The simplest and most logical fate for any administered NPs is the incorporation by macrophages. This suggests that the future perspectives for the use of NPs to target and treat macrophages is guaranteed by the nature of these phagocytic cells. In fact, many efforts in the field of pharmaceutical technology are devoted nowadays to fine-tune the NP surface to skip the macrophage uptake and to target other cell types. These facts together with the growing interest for combined immunological therapies opens new avenues for the design of novel therapeutic systems at the nanoscale to modulate macrophages in different clinical scenarios.

## Figures and Tables

**Table 1:** Nanoparticle-based strategies to modulate macrophages in chronic inflammation.

Disease	Type of NP	Payload	Model	Effects on Macrophages	Outcome	Ref.
Rheumatoid Arthritis	Tuftsin-modified non-condensing alginate-based NPs	pDNA IL-10	In vivo	Induce IL10 expression	Polarization to M2	[19]
	MFC-MSNs	-	In vivo	Scavenge ROS and produce O_2_	Polarization to M2	[20]
	Chitosan/siRNA nanoparticles	TNFα siRNA	In vivo	Reduce TNFα expression	M1 depletion	[21]
	Folate–Chitosan DNA nanoparticles	IL-1Ra Gene	In vivo	Reduce IL-1 expression		[22]
	FA-conjugated G5 dendrimers	Methotrexate	In vivo	Reduce cell proliferation and induce apoptosis	M1 depletion	[23]
	pH-responsive nanocarriers	Methotrexate	In vivo			[24]
	Polymeric nanoparticles	Dexamethasone and naproxen	In vitro	Prevent IL-12 expression	Polarization to M2	[25]
	FA modified ROS-responsive nanoparticles	Dexamethasone	In vivo	Interfere in the iRhom2-TNF-α-BAFF signaling pathway		[26]
Inflammatory Bowel Disease (IBD)	Mannosylated nanoparticles	TNFα siRNA	In vitro Ex vivo	Reduce TNFα expression	M1 depletion and colitis attenuation	[27]
	NPs grafting Fab’ portion of the F4/80 antibody	TNFα siRNA	In vitro	Reduce TNFα expression		[28]
	Termed thioketal nanoparticles	TNFα siRNA	In vivo	Reduce TNFα expression		[29]
	GC PLGA NPs	TNFα siRNA	In vitro In vivo	Reduce TNFα expression		[30]
	Nanoparticles-in-microsphere oral system (NiMOS)	TNFα siRNA	In vivo	Reduce TNFα expression		[31]
		Cyclin D1 siRNA		Reduce Cyclin D1 expression		
	b1,3-D-glucan- particles (GeRPs)	Map4K4 siRNA	In vivo	Reduce Map4K4 expression		[32]
Atherosclerosis	Mannose functionalized dendrimer NPs	SR-A siRNA	In vivo	Reduce LDL uptake	Plaque regression	[33]
		LXR ligand		Stimulate cholesterol efflux		
	HA-coated cell-penetrating peptide nanocomplexes	LOX-1 siRNA	In vivo	Reduce LDL uptake	Plaque regression	[34]
	Lipid nanoparticle	CCR2 siRNA	In vivo	Reduce CCR2 expression	Reduce macrophage recruitment	[35]
	EGFP-EGF1-conjugated PLGA nanoparticles	CCR2 shRNA	In vitro	Reduce CCR2 expression		[36]
	S2P-conjugated DSPE-PEG NPs	CaMKIIγ siRNA	In vivo	Unblock macrophage efferocytosis	Plaque stabilization	[37]
	Single-walled carbon nanotubes	TPI	In vitro	Stimulate efferocytosis		[38]

**Table 2:** Nanoparticle-based strategies to modulate macrophages for tissue repair.

Disease	Type of NP	Payload	Model	Effects	Outcome	Reference
Ischemic injury in muscle	AuNPs	IL-4	In vivo	Polarization to M2	Muscle regeneration	[54]
Periodontitis	AuNPs	-	In vivo	Polarization to M2	Periodontal tissue regeneration	[55]
Bone injury or defect	TiO_2_	-	In vitro	Polarization to M2	Bone regeneration	[56]
	Mesoporous silica NPs (MSNs)	-	In vitro	Enhance the osteogenic differentiation of BMBMs		[57]
	Copper Mesoporous silica NPs (MSNs)	-	In vitro			[58]
	Alendronate conjugated GNPs	-	In vivo	Inhibit BMDMs differentiation to osteoclasts		[59]
Myocardial infarct (MI)	PS-presenting liposomes	-	In vivo	Macrophage transition to reparative state	Myocardial infarct repair	[60]
	Hyaluronan-sulfate (HAS)-Ca^2+^ NPs	miRNA-21	In vivo	Macrophage transition to reparative state		[61]
	Polyketal (PK3) nanoparticles	miRNA	In vitro In vivo	Reduce Nox_2_ expression, leading to downregulation of inflammation		[62]
	Lipidoid nanoparticles (LNPs)	IRF5 siRNA	In vivo	Attenuation of M1 macrophage polarization		[63]
	PLGA NPs	Pioglitazone	In vivo	NF-κB inhibition, leading to inflammation downregulation		[64]
Chronic liver injury	MTC conjugate nanoparticles	TNFα siRNA	In vivo	Reduce TNFα expression	Reduce liver injury and fibrosis	[65]
	PLGA NPs	SYK pathway inhibitor	In vitro In vivo	SYK inhibition		[66]
	Liposomes	Dex	In vivo	Anti-inflammatory polarization of macrophages		[67]
	PS-modified nanostructured lipid carriers (mNLCs)	Curcumin	In vivo	Anti-inflammatory effects		[68]
	Dendrimer-Graphene nanostars	Plasmid expressing MMP9	In vitro	Overexpression of MMP9 which lead to macrophage transition to reparative state		[69]

**Table 3:** Nanoparticle-based strategies targeting tumor-associated macrophages for cancer diagnosis and therapy.

Cancer Diagnosis				
Type of NP	Diagnostic Strategy	Cancer Model	TAM Targeting	References
AMTA680	Fluorescence imaging and MRI	Soft tissue sarcoma, lung carcinoma and colon adenocarcinoma	Accumulated in TAM rich areas	[88]
Iron oxide NP	MRI	Breast		[89]
^89^Zr-modified rHDL	PET			[94]
Silica-coated iron oxide NP	Fluorescence imaging	Glioblastoma		[97]
^64^Cu-loaded mannosylated liposomes	PET	Pulmonary adenocarcinoma		[96]
^64^Cu-labeled polyglucose NPs		Breast		[95]
Perfluorocarbon	MRI	Breast		[93]
Ferumoxytol iron oxide NP		Anaplastic thyroid cancer		[90]
		Melanoma		[91]
**Cancer Therapy**				
**Therapeutic Approach**	**Type of NP**	**TAM Targeting**	**Cancer Model**	**References**
Macrophage recruitment inhibition	Cationic polymeric NPs	CCR2	Breast	[98]
	Ultrasmall cooper NPs		Pancreatic ductal adenocarcinoma	[99]
TAM depletion	PEI-SA nanomicelles	CSF-1R	Pancreatic	[100]
	DMCP and cholesterol oleate		Melanoma	[101]
	PLGA		Melanoma	[102]
	Hydroxyl dendrimer NPs		Glioblastoma	[103]
	Dextran grafted poly(histidine) copolymer + erythrocyte/cancer cell membrane hybrid		Breast	[104]
	Red fluorescent polymeric micelle		Pulmonary melanoma	[105]
	Self-assembled colipids (DSPE, PEG and PC)		Melanoma and breast cancer	[106]
Blocking macrophage “do not eat me” signal	Magnetic NPs	CD47	Melanoma and triple negative breast cancer	[107]
	Bismuth selenide NPs		Breast	[108]
	Self-assembled colipids (DSPE, PEG and PC)	SHP2	Melanoma and breast cancer	[106]
Functional TAM reeducation	PEG and trimethyl chitosan modified with mannose	VEGF PIGF	Breast	[109]
	Gold core NPs decorated with thiolated-PEG-COOH polymer	VEGF	Lung	[110]
	Hyaluronic acid-poly (ethylenimine) NPs	miR-125b		[111]
	Anionic magnetic NPs		Breast	[112]
	Ferumoxytol iron oxide NPs	TLR3	Melanoma and lung metastasis	[113]
	Iron oxide NPs	P13K and mannose receptor	Breast	[114]
		TLR7		[115]
	Human ferritin heavy chain nanocages	TLR		[116]
	Phosphatidylglycerol nanocomplexes with stearic acid	BTK and Siglec-1	Sarcoma	[117]

## Data Availability

Not applicable.
